# Right ventricular lead migration to the abdominal cavity: a case report of an unexpected journey

**DOI:** 10.1093/ehjcr/ytaf179

**Published:** 2025-04-10

**Authors:** Ibrahim Antoun, Ayman Helal, Azhar Farooqui, Mohsin Farooq, Mohammad El-Din

**Affiliations:** Department of Cardiology, Kettering General Hospital, Kettering NN16 8UZ, UK; Department of Cardiovascular Sciences, University of Leicester, Leicester LE1 7RH, UK; Department of Cardiology, Kettering General Hospital, Kettering NN16 8UZ, UK; Department of Cardiology, Kettering General Hospital, Kettering NN16 8UZ, UK; Department of Cardiology, Kettering General Hospital, Kettering NN16 8UZ, UK; Department of Cardiology, Kettering General Hospital, Kettering NN16 8UZ, UK

**Keywords:** Pacemaker lead, Complication, Lead perforation, Colon, Case Report

## Abstract

**Background:**

Lead perforation, though an uncommon complication of cardiac device implantation, is associated with significant morbidity, especially when leads migrate to extracardiac structures. Lead migration into the abdominal cavity is exceedingly rare, and management in such cases can be complex.

**Case Summary:**

We present the case of an 82-year-old woman with known dementia who underwent single-chamber pacemaker implantation for symptomatic Mobitz Type II atrioventricular (AV) block. Two weeks post-implantation, the nursing home staff observed that the patient had bradycardia. Electrocardiogram on hospital admission demonstrated recurrence of Mobitz Type II AV block. Pacing checks confirmed there was no lead sensing. Imaging studies confirmed that the right ventricle lead had perforated the myocardium, passed through the diaphragm, and migrated into the abdominal cavity near the colon. The case was discussed in a multidisciplinary team. The final clinical decision was to extract the displaced lead to avoid the risk of further intra-abdominal organ perforations and the risk of developing pericardial effusion. A new lead was successfully implanted in the septal position, with subsequent follow-up showing stable pacing function. The patient received an extended course of antibiotics and made an uneventful recovery leading up to discharge.

**Discussion:**

This case underscores the importance of prompt recognition and a multidisciplinary approach to managing instances of rare lead migration, particularly in elderly, frail patients. Careful imaging and risk assessment helped guide the decision-making process, balancing the risks of lead extraction against potential complications.

**Learning points**

• Rare cases of pacemaker lead migration to extracardiac structures require early recognition and a multidisciplinary approach to balance procedural risks and optimize outcomes, especially in elderly or frail patients.

## Introduction

Lead perforation is an uncommon but serious complication of cardiac pacemaker implantation, with significant consequences that range from minor local tissue injury to life-threatening events such as pericardial effusion, tamponade, or organ perforation.^1^ The right ventricular (RV) pacing lead is prone to dislodgement and perforation, often occurring shortly after implantation. Lead dislodgement usually presents with symptoms of lead malfunction, such as loss of capture or clinically significant symptoms like chest pain, shortness of breath, or hypotension.^2^ The incidence of lead perforation has been reported as 0.8% among patients with newly implanted pacemakers,^1^ with risk factors including active fixed leads, lower body mass index and higher fluoroscopy times, females and increased age.^3,4^

Uncommonly, RV leads can perforate extracardiac structures, including the diaphragm or abdominal organs, if migration progresses. However, migration to the abdominal cavity is exceedingly rare, with few cases documented in the literature. This scenario can pose significant clinical challenges due to the risk of infection, potential for bowel injury, and complications related to the extraction of the dislodged lead. Management typically requires a multidisciplinary approach involving electrophysiology, cardiothoracic surgery, and, at times, gastrointestinal specialists to address the risks associated with lead extraction and potential gastrointestinal perforation.

In this report, we discuss the case of an 82-year-old woman who presented with symptomatic bradycardia and subsequently underwent single-chamber pacemaker implantation. While her initial post-procedural course was uncomplicated, her pacemaker lead later migrated, perforating through the RV wall and diaphragm to eventually lodge within the abdomen. This case underscores the importance of vigilance in follow-up imaging and highlights the complexities of managing atypical lead dislodgement in a frail, elderly patient.

## Summary figure

**Figure:**
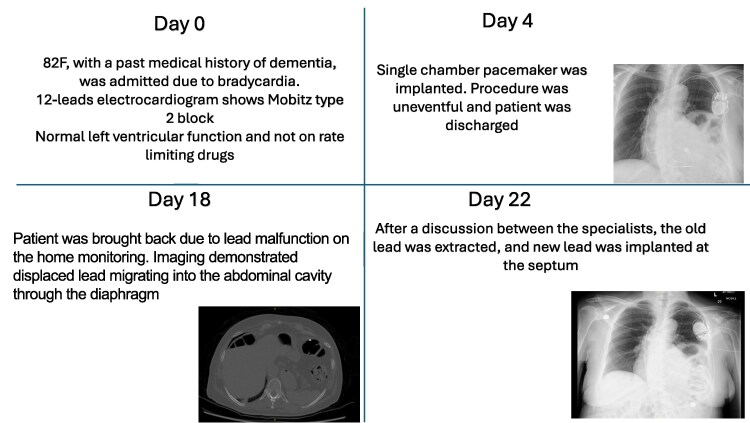


## Case presentation

Our patient is an 82-year-old female who presented to the emergency department (ED) due to shortness of breath. She has a background history of dementia, hypertension and chronic kidney disease (CKD) Stage 4. She has no lasting power of attorney or an advanced directive regarding health issues. She normally lives in a care home. Her cardiovascular examination yielded a clear chest and normal heart sounds but bradycardic at 40 beats per minute. She was not on any rate-limiting drugs, and her 12-leads electrocardiogram (ECG) confirmed a 2:1 Mobitz Type II atrioventricular (AV) block. Her QRS duration was normal, and her transthoracic echocardiogram demonstrated a preserved left ventricular systolic function. Admission blood results included haemoglobin: 11.8 g/dL, white cell count: 8.2 × 10^9^/L, C-reactive protein: 12 mg/L, creatinine: 225 μmol/L (baseline consistent with CKD Stage 4), and electrolytes: normal limits, thyroid function: normal limit.

**Figure 1:**
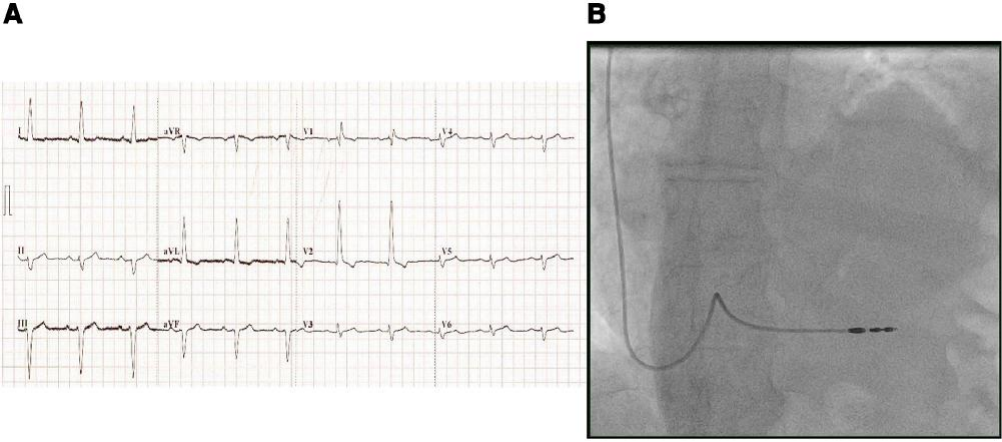
*(A)* demonstrates the anteroposterior CXR after the first device implant, ruling out pneumothorax and showing a satisfactory lead position at the apical septum. *(B)* demonstrates a CXR 1 year ago showing a similar left hemidiaphragm elevation.

**Figure 2:**
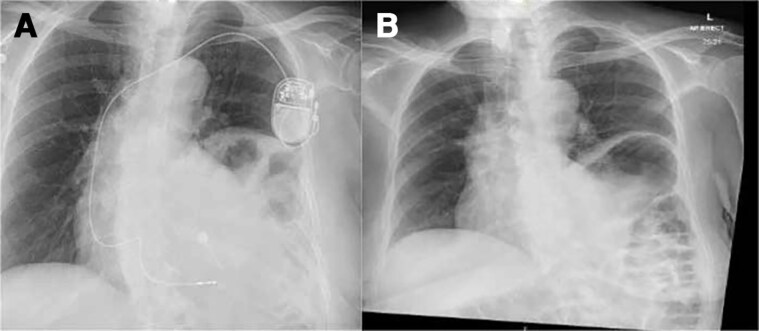
*(A)* 12 leads ECG after the first implant demonstrating sinus rhythm without needing pacing. *(B)* RAO fluoroscopic view after the first implant.

Due to her frailty, she went on to have a single-chamber VVI pacemaker implantation, a decision taken by the cardiac devices consultant after considering her functional baseline and in consultation with the next of kin (NoK) after the patient was deemed not to have the mental capacity to make that decision. The procedure was uneventful, and the lead was placed at the apical position using a Biotronik Solia S60 active fixation lead and a Biotronik Enitra 6 SR pacemaker. The pacemaker and the lead are not known to migrate or perforate the myocardium more commonly than other models. The procedure was done under post-ero-anterior and right anterior oblique (RAO) fluoroscopic views (*Figure 1B*). The injury current was positive when the lead was deployed. Left anterior oblique (LAO) and right anterior oblique (RAO) fluoroscopy views were not recorded during the first procedure. The ECG following the first implant showed sinus rhythm without heart blocks without pacing morphology (*Figure 1A*). The post-procedure pacing checks were satisfactory, and the post-procedure chest X-ray (CXR) did not identify any pneumothorax with the RV lead in the radiologically appropriate location (*Figure 2A*). It also identified an elevated left hemidiaphragm, present on a previous CXR performed 1 year ago (*Figure 2B*). She was discharged to her care home following an uneventful recovery.

**Figure 3:**
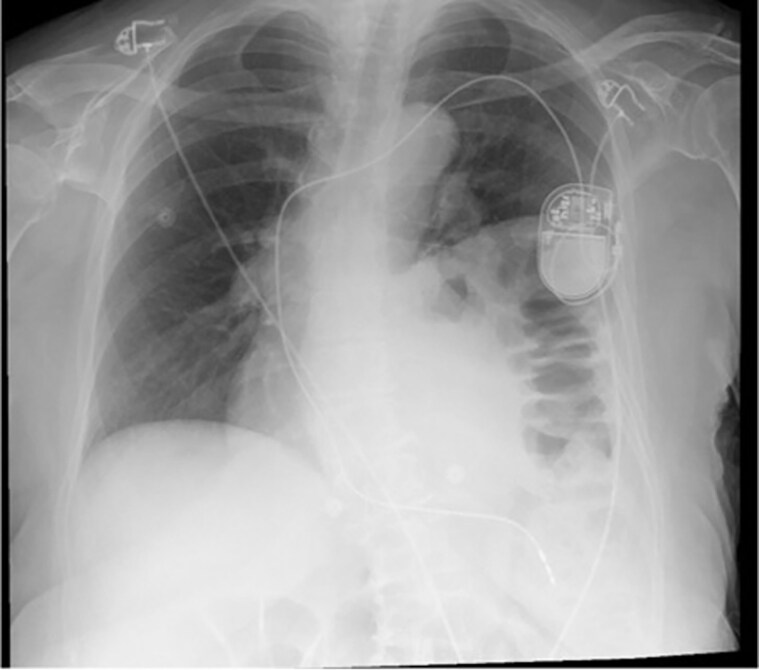
CXR on the second admission 2 weeks after the implant demonstrating pacemaker-lead migration to below the left hemidiaphragm on top of the colon.

**Figure 4:**
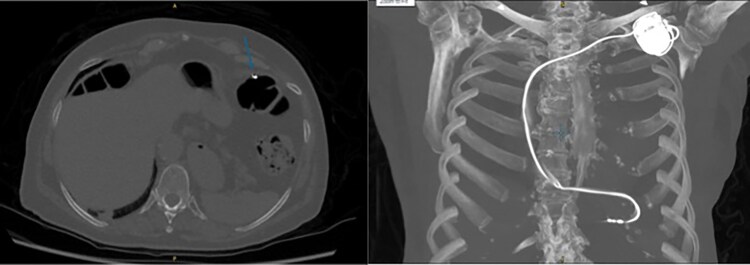
CT scan demonstrating displaced pacemaker lead into the abdominal cavity.

Two weeks after the initial implantation, her nursing home staff noticed the patient had a slow heart rate—home cardiac device monitoring identified under-sensing in unipolar and bipolar vectors. There was no capture at 7.5 volts (V). She was brought to the ED, and her ECG confirmed a 2:1 Mobitz Type II AV block. She was hemodynamically stable and asymptomatic. Repeated CXR (*Figure 3*) demonstrated a displaced pacing lead below the left diaphragm overlying the colon. A bedside echocardiogram ruled out pericardial effusion; however, it could not accurately locate the tip of the pacing lead. An urgent computed tomography (CT) of the chest and abdomen was arranged, which demonstrated a displaced pacing lead exiting the right ventricle (RV) and diaphragm and resting inside the abdominal cavity anterior to the transverse colon with a possibility of perforation (*Figure 4*).

**Figure 5:**
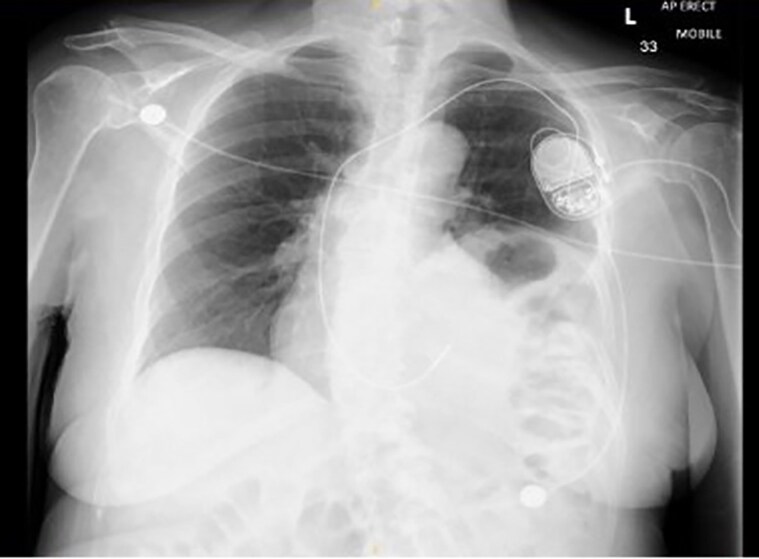
CXR following the new lead implantation at the septal position demonstrating satisfactory lead position and no pneumothorax.

Furthermore, the CT did not demonstrate a cause of the raised left hemidiaphragm. Clinically, the patient had a soft abdomen, with normal bowel sounds, and no evidence of guarding or rigidity. Therefore, there was no clinical evidence of perforation of abdominal viscera. The case was discussed in the multidisciplinary team meeting (MDT), which involved three cardiac devices specialists, a cardiac surgeon, a gastrointestinal surgeon and a microbiologist. The proposed options included extraction without further therapy, lead extraction with a new lead implantation at the septum, or leaving the old lead and implantation of a new lead at the septum. The second option was proposed as the patient remained to have a pacing indication, and there was uncertainty about the ramifications of leaving the pacing lead in the abdominal cavity. The NoK was informed about this and the proposed MDT plan, and she was happy to agree with what the medical team believed was the best suitable option for the patient’s interest. The procedure occurred 3 weeks after the initial implant, and an active fix RV lead was planted in the septal position. The old lead was extracted without complications and sent for culture (*Figure 5*), which grew staphylococcus hominis, a known skin contaminant. Antibiotic cover was given before and after the procedure guided by the microbiology team. The CXR following the procedure showed a well-placed RV lead in the septum (*Figure 5*), and the echocardiogram ruled out any effusion. The patient was covered with piperacillin and tazobactam for 5 days after involving the microbiology team with satisfactory inflammatory markers and discharged home. A pacing check 6 weeks after the implant showed satisfactory pacing checks with an R wave amplitude of 5.3 millivolts (mV), a threshold of 0.9 V, and a pulse width of 0.4 milliseconds. Parameters were similar when checked again 6 weeks later. The patient is currently on annual pacemaker follow-up as per the hospital protocol.

## Discussion

Pacemaker lead perforation is a rare but potentially serious complication of cardiac device implantation, with risks ranging from lead malfunction to life-threatening complications such as pericardial effusion or organ injury.^1^ While most cases involve minor myocardial penetration, extracardiac migration—particularly into the abdominal cavity—is exceedingly rare. This case highlights the importance of early recognition, appropriate imaging, and a multidisciplinary approach to guide management decisions, especially in frail elderly patients. Risk factors include advanced age and female sex, which were present in our patient.^4^ Elderly patients with frailty, as in our case, often have reduced physiological reserves, making them less resilient to invasive interventions and more vulnerable to complications. For this reason, clinical teams frequently face a dilemma: the decision between surgical extraction of the perforated lead, which has potential risks, and leaving the lead *in situ*, potentially leading to chronic irritation or infection.

Uncommonly, RV leads can perforate extracardiac structures, including the diaphragm or abdominal organs, if migration progresses. However, a pacemaker lead migration to the abdominal cavity is exceedingly rare, without previous reports in the literature. This scenario can pose significant clinical challenges due to the risk of infection, potential for bowel injury, and complications related to the extraction of the dislodged lead. In our case, CT imaging played a key role in accurately identifying the position of the dislodged lead and confirming its location within the abdomen. CT was proven superior to plain X-ray in diagnosing perforated leads, and it aids in planning the safest and most effective intervention strategy, minimizing the risk of further complications such as in our case.^5,6^

The absence of pericardial effusion following the perforation of the pacing lead is noteworthy and can be attributed to several physiological and anatomical factors. Typically, lead perforation can lead to pericardial effusion due to the disruption of the pericardial space; however, in this instance, the perforation did not result in significant fluid accumulation. One possible explanation is that the perforation may have been small or limited in extent, allowing the pericardial space to remain intact without substantial blood or fluid leakage into the surrounding area.^7,8^ Moreover, the pericardium is a fibrous sac that can sometimes effectively seal small perforations, preventing effusion development. This phenomenon has been observed in other cases where minor perforations did not lead to effusion or cardiac tamponade, suggesting that the body's compensatory mechanisms can sometimes mitigate the consequences of such injuries.^9^ The echocardiographic assessment performed in this case, which ruled out effusion, supports the notion that the perforation was not severe enough to elicit a significant inflammatory response or fluid accumulation in the pericardial cavity.^10^ We propose multiple measures to follow to avoid this complication. In general, active fixation leads should be avoided at the RV apex, and passive leads should be used instead if an apical position is considered to minimize the risk of perforation. For elderly or frail patients with an increased risk of lead perforation, operators should consider septal lead placement over apical placement, as septal positioning may reduce the risk of perforation by avoiding thinner myocardial regions.^4^ Incorporating fluoroscopy, including LAO and RAO views, is advised to confirm proper lead placement and minimize perforation risks. Further steps to avoid this complication include ensuring that active fixation leads are appropriately secured without excessive screwing, which can increase the risk of myocardial injury and perforation, Emphasising close monitoring of device performance, especially in the first few weeks post-implantation, to promptly identify signs of lead dislodgement or malfunction, Identifying patients at higher risk (elderly females with low body mass index or significant comorbidities such as our patient) and tailoring implantation strategies, such as selecting passive fixation leads when clinically appropriate.^4^

This case stresses the importance of comprehensive follow-up for frail elderly patients with new pacemaker implants, especially those at higher risk of complications, including lead perforations. It also highlights the value of an MDT in managing rare and complex lead perforations, utilising expertise from various subspecialists to achieve optimum outcomes.

## Data Availability

Data regarding this case report is available on request from the corresponding author.

## Lead author biography

Graduated from University of Aleppo with MD degree in 2016. UK licenced physician and a member of the Royal College of Physicians in the United Kingdom in 2021. Completed his PhD in Electrophysiology in the University of Leicester in 2023. Currently works as a Cardiology trainee in East Midlands deanery and an honorary research fellow at the University of Leicester.

**Consent:** The authors confirm that informed consent was obtained from the patient’s NoK since the patient did not have the capacity to consent.

**Funding:** The procedure was carried out in an NHS institution. No external funding was required. The authors consent that there is no conflict of interest to declare.
